# The relationship between family health, stress, and self-efficacy on depression among university students: a large-scale national cross-sectional study

**DOI:** 10.3389/fpubh.2025.1625269

**Published:** 2025-07-31

**Authors:** Zhihao Zhang, Wenyue Liu, Chen Zhang, Lezhong Sun

**Affiliations:** ^1^Faculty of Education, Universiti Kebangsaan Malaysia, Bangi, Malaysia; ^2^Shandong Vocational University of Foreign Affairs, Weihai, Shandong, China

**Keywords:** family health, stress, self-efficacy, university students, depressive symptoms

## Abstract

**Background:**

Depression is a prevalent mental disorder globally, significantly impacting university students who face unique challenges such as academic and family pressures. This study investigates the prevalence of depressive symptoms among university students, examining the mediating role of stress in the relationship between family health and depressive symptoms, and the moderating role of self-efficacy in these relationships during COVID-19.

**Methods:**

A survey was conducted across various regions in China, collecting data from 1,193 university students. The instruments used included the Patient Health Questionnaire-9 to assess depressive symptoms, the Family Health Scale-Short Form to measure family health, the Subjective Life Stress Scale to evaluate stress levels, and the New General Self-Efficacy Scale to assess self-efficacy.

**Results:**

The results indicated a 26.8% prevalence of depressive symptoms among participants. Family health was found to negatively correlate with depressive symptoms, with stress mediating this relationship. Additionally, self-efficacy moderates both the direct and indirect effects of family health on depressive symptoms.

**Conclusion:**

This study underscores the need to develop comprehensive mental health strategies that consider the combined associations of family health, stress management, and self-efficacy with depressive symptoms among university students. It is particularly important to provide more effective support and interventions for the mental health of university students in the post-pandemic era.

## 1 Introduction

Depression is one of the most prevalent mental disorders globally, affecting millions of individuals annually and emerging as a leading cause of disability and premature mortality (1). In 2021, approximately 27.96 million young people aged between 20 and 24 years were afflicted with depression worldwide (2). University students, a predominant demographic within this age range, face multiple challenges including academic stress, employment stress, and social restrictions, leading to an unusually high prevalence of depression (3), particularly during the COVID-19 pandemic (4). Depressive symptoms not only hinder academic and social functioning but also encompass severe mental health issues such as self-harm and suicide (5–7). Consequently, early identification of factors associated with depressive symptoms among university students and investigation into the potential mechanisms of these factors on depressive symptoms are of paramount importance for the effective prevention and treatment of depression.

According to Bronfenbrenner’s Ecological Systems Theory, the family, as the innermost and most influential microsystem (8), plays a pivotal role in the early stages of psychological development during adolescence and young adulthood (9, 10). Family health, as a core indicator of the quality of the family environment, reflects the family’s functional capacity in areas such as emotional support, communication, behavioral norms, and role allocation (11). Research has shown that individuals who benefit from higher levels of family involvement in health-related matters are more likely to receive health-related support and care from family connections. This support helps enhance their health literacy, thereby reducing the likelihood of mental health issues (12). Moreover, numerous studies have explored the relationship between family health and adverse psychological symptoms, such as depression and anxiety, indicating that poor family health increases the probability of maladaptive psychological adjustment in adolescents and undermines their overall well-being (13–15).

An increasing body of research indicates that perceived stress is one of the key predictors of depressive symptoms, with high levels of perceived stress often being significantly associated with the exacerbation of depressive moods (16). The Stress-Vulnerability Model suggests that the onset of psychological symptoms results from the interaction between external stressors and individual vulnerability (17). When an individual faces high levels of life stress and lacks sufficient internal regulatory resources, they are more prone to experiencing psychological distress (18). During the university years, individuals face multiple life challenges, including academic pressure, interpersonal issues, career uncertainty, and family expectations (19). It is important to note that the influence of the family of origin on psychological development is enduring. Even as university students achieve physical independence, their emotional response patterns, sense of self-worth, and interpersonal tendencies continue to be shaped and influenced by early family experiences (20). Prolonged exposure to a family environment characterized by low support and high conflict can impair an individual’s cognitive regulation of stress, intensify negative emotional responses, and contribute to the development of depressive tendencies (19). Conversely, a healthy family environment not only provides stable emotional support but also enhances an individual’s coping abilities, reducing their sensitivity to stress. Thus, it may be inferred that the relationship between family health and depressive symptoms in university students may be mediated by perceived stress.

In addition to external environmental factors, an individual’s internal psychological resources play a crucial role in the onset and development of depressive symptoms. Self-efficacy, a core concept in Bandura’s Social Cognitive Theory, refers to an individual’s belief and confidence in their ability to successfully complete specific tasks or effectively cope with particular situations (21). Self-efficacy not only influences an individual’s motivation, behavior, and emotional response patterns but also serves as a vital internal resource when facing stress and challenges.

A growing body of research has shown that higher levels of self-efficacy significantly reduce the risk of developing depressive and anxiety symptoms. For example, studies by PK Maciejewski and PK Sharma found that individuals with high self-efficacy are more likely to employ proactive coping strategies, such as problem-solving and seeking social support, when faced with stress, thereby exhibiting lower levels of depressive and anxiety symptoms (22–24). Furthermore, higher self-efficacy contributes to enhancing an individual’s psychological resilience, enabling them to adapt more effectively to common challenges in university life, such as academic stress and interpersonal difficulties, and thereby reducing the likelihood of psychological distress (25, 26). However, while self-efficacy is a critical internal protective factor, its specific role in moderating the relationship between family health, perceived stress, and depressive symptoms in university students remains insufficiently explored and requires further investigation.

Based on Bronfenbrenner’s Ecological Systems Theory, the Stress-Vulnerability Model, and Bandura’s Social Cognitive Theory (8, 17, 21), this study constructs a moderated mediation model with stress as the mediator and self-efficacy as the moderator, to explore the relationship between family health and depressive symptoms in university students. Specifically, this study aims to (1) estimate the prevalence of depressive symptoms among Chinese university students; (2) investigate whether stress mediates the association between family health and depressive symptoms; and (3) determine whether self-efficacy moderates this mediating pathway. The results are expected to inform the identification of high-risk groups and to offer theoretical and practical insights for the prevention and intervention of depression in this population, thereby contributing to more effective strategies for promoting mental health among university students.

## 2 Methods

### 2.1 Participants

The data utilized in this study were derived from the Psychology and Behavior Investigation of Chinese Residents (PBICR) survey (27, 28), conducted in China from July 10 to September 15, 2021, during the COVID-19 pandemic. This cross-sectional study employed a multistage sampling method. Based on data from the Seventh National Population Census of the People’s Republic of China in 2021, a sample of residents was selected from 120 cities using quota sampling, with quotas determined by gender, age, and urban–rural distribution. Participants were required to meet the following inclusion criteria: (1) holding Chinese nationality; (2) voluntarily participating in the study; (3) being able to independently complete the online questionnaire or with assistance from research personnel; and (4) being capable of understanding all items on the questionnaire. At least one interviewer or one interview team was recruited in each city, with each interviewer responsible for collecting 30 to 90 questionnaires and each interview team responsible for collecting 100 to 200 questionnaires. A total of 11,709 questionnaires were distributed, and 11,031 valid questionnaires were recovered, yielding an effective response rate of 94.21%. In this study, a total of 1,193 enrolled university students aged 19–25 was included, with no missing data.

### 2.2 Measures

#### 2.2.1 Sociodemographic characteristics

Data on eight variables were collected, including gender, location, education level, monthly income per capita in family (Yuan), household debt, living alone, smoking, BMI, and frequency of cell phone use. The detailed measurement methods for these variables have been documented in previous studies (27).

#### 2.2.2 Family health

The Family Health Scale–Short Form (FHS-SF), originally developed by Crandall and Weiss-Laxer (29), was employed to assess the family health status of university students. With the original authors’ permission, Wang et al. translated the scale into Chinese, which demonstrated good reliability and validity and is suitable for assessing family health in Chinese populations (30). The FHS-SF comprises 10 items with high factor loadings and weights across four dimensions: family social and emotional health processes, family health lifestyle, family health resources, and external social support. Each item is rated on a 5-point Likert scale, with items 6, 9, and 10 being reverse-scored. Higher total scores indicate a higher level of family health. The FHS-SF demonstrated good reliability in this study, with a Cronbach’s alpha of 0.85.

#### 2.2.3 Depression

The Patient Health Questionnaire-9 items (PHQ-9) was used to assess depressive symptoms in university students. This scale, developed by Spitzer and colleagues (31), measures the actual experiences of respondents related to depression over the past 2 weeks. The scale consists of nine items that evaluate the following aspects: reduced interest, depressed mood, sleep disorders, fatigue, eating disorders, feelings of worthlessness, difficulty concentrating, psychomotor retardation, and suicidal symptoms. Each item is rated using a 4-point Likert scale, ranging from “never” (0 points) to “nearly every day” (3 points). The total score is calculated by summing the scores of all items. The scale categorizes total scores as follows: 0–4 indicating no depression, 5–9 suggesting possible mild depression, 10–14 indicating possible moderate depression, 15–19 indicating possible moderately severe depression, and 20–27 indicating possible severe depression. The higher the total score, the more likely the individual is to have depression. In this study, PHQ-9 total score of ≥10 was considered as potentially indicating clinical depressive symptoms (3, 32). In this study, PHQ-9 demonstrated excellent internal consistency, with a Cronbach’s alpha of 0.95.

#### 2.2.4 Stress

The Subjective Life Stress Scale was used to survey the stress levels of university students. This scale was developed by members of the PBICR according to standard questionnaire development procedures and is designed for self-assessment of personal stress. The questionnaire comprises three items that inquire about the respondent’s “ability to handle stress,” “level of stress in life (including family and academics) over the past 2 weeks,” and “level of stress in life (including family and academics) over the past year.” Each item is scored using a 6-point Likert scale, with the scoring based on the respondent’s perceived level of stress. Item 1 is scored from “I am able to get rid of stress” (1 point) to “stress has been bothering me for a long time” (6 points), while items 2 and 3 are scored from “no stress” (1 point) to “extreme stress” (6 points). The total score is the sum of all items, with higher scores indicating that the respondent more strongly self-perceives stress. The Subjective Life Stress Scale showed good reliability in this study, with a Cronbach’s alpha value of 0.82.

#### 2.2.5 Self-efficacy

The New General Self-Efficacy Scale (NGSES) was used to assess the self-efficacy of university students. This scale was adapted by Chen G (33) from the General Self-Efficacy Scale (34). The NGSES consists of 8 items, all of which are positively scored. Each item is scored using a 5-point Likert scale, ranging from “strongly disagree” (1 point) to “strongly agree” (5 points). The total score is the sum of all items, with higher scores indicating better self-efficacy. The NGSES exhibited excellent reliability in this study, with a Cronbach’s alpha of 0.95.

### 2.3 Statistical analysis

To present the baseline characteristics of the participants, continuous variables were expressed as means ± standard deviations (*SD*), and between-group differences were examined using chi-square tests and independent samples *t*-tests. The correlation between study variables (depressive symptoms, family health, stress, and self-efficacy) was analyzed using Pearson’s correlation analysis. The mediation and moderated mediation models (35) were analyzed using the PROCESS macro in SPSS 27.0. The 95% confidence intervals (CI) for the adjusted biases were calculated through 5,000 bootstrap resamples. Initially, Model 4 was used to test whether stress mediated the relationship between family health and depressive symptoms. If the 95% CI for the indirect effect did not include zero, it indicated a significant mediating effect. Subsequently, Model 59 was employed to test a moderated mediation model, examining whether self-efficacy moderated both the direct and indirect associations between family health and depressive symptoms. Again, if the 95% CI for the interaction did not include zero, a significant moderated mediation effect was established. Conditional effects and confidence intervals were plotted using the Johnson-Neyman technique (36). Additionally, all models controlled for covariates (gender, location, monthly income per capita in family (Yuan), household debt, living alone, smoking, BMI, frequency of cell phone use), and the study variables were standardized.

## 3 Results

### 3.1 Sociodemographic characteristics and depressive symptoms

Among the 1,193 university students surveyed, 21.8% reported depressive symptoms. As shown in Table 1, depression was more common among male students than female students. Students who lived alone, smoked, or used their cell phones frequently were also more likely to experience depressive symptoms. Moreover, students with depressive symptoms reported lower levels of self-efficacy, poorer family health, and higher levels of stress. All reported differences were statistically significant at the *p* < 0.05.

**Table 1:** Sociodemographic characteristics and the distribution of depressive symptoms.

Characteristics	Total	Depression		*p*	*χ^2^*/*t*
		No	Yes		
*n* (%)	1,193	933 (78.2)	260 (21.8)		
Gender				0.035	4.432
Male	483 (40.49)	363 (75.16)	120 (24.84)		
Female	710 (59.51)	570 (80.28)	140 (19.72)		
Location				0.278	1.175
City	831 (69.66)	657 (79.06)	174 (20.94)		
Country	362 (30.34)	276 (76.24)	86 (23.76)		
Monthly income per capita in family (Yuan)				0.221	3.023
≤3,000	420 (35.21)	329 (35.26)	91 (35.00)		
3,001–7,500	539 (45.18)	412 (44.16)	127 (48.85)		
>7,500	234 (19.61)	192 (20.58)	42 (16.15)		
Household debt	518 (43.42)	414 (44.37)	104 (40.00)	0.208	1.583
Living alone	80 (6.71)	47 (5.04)	33 (12.69)	<0.001	19.045
Smoking	38 (3.19)	22 (2.36)	16 (6.15)	0.002	9.501
BMI				0.379	
<18.5	250 (20.96)	190 (20.36)	60 (23.08)		
18.5 to 24	768 (64.38)	612 (65.59)	156 (60.00)		
24 to 28	139 (11.65)	103 (11.04)	36 (13.85)		
≥28	36 (3.02)	28 (3.00)	8 (3.08)		
Frequency of cell phone use				<0.001	108.143
Never	12 (1.01)	10 (1.07)	2 (0.77)		
Occasionally	20 (1.68)	10 (1.07)	10 (3.85)		
Sometimes (2–3 days/week)	127 (10.65)	69 (7.40)	58 (22.31)		
Often (4–5 days/week)	131 (10.98)	76 (8.15)	55 (21.15)		
Almost daily (6–7 days/week)	903 (75.69)	768 (82.32)	135 (51.92)		
Depression	6.88 ± 6.01	4.35 ± 3.19	15.98 ± 4.84	<0.001	−45.868
Self-efficacy	28.73 ± 5.46	28.94 ± 5.22	27.99 ± 6.20	0.024	2.263
Family health	37.85 ± 6.49	38.78 ± 6.52	34.51 ± 5.12	<0.001	11.159
Stress	9.26 ± 3.60	8.71 ± 3.44	11.25 ± 3.47	<0.001	−10.471

Variables are presented as mean ± SD or *n* (%).

### 3.2 Bivariate correlations among all the variables

Table 2 indicated that depressive symptoms were negatively correlated with family health and self-efficacy, while positively correlated with stress. Family health was positively correlated with self-efficacy and negatively correlated with stress. Stress showed negative correlations with both family health and self-efficacy. All correlations were statistically significant at the *p* < 0.05.

**Table 2:** Correlations of the studied variables (*n* = 1,193).

Variable	1	2	3
Depressive symptoms			
Family health	−0.269**		
Stress	0.404**	−0.273**	
Self-efficacy	−0.059*	0.499**	−0.207**

** *p* < 0.01, *** *p* < 0.001.

### 3.3 Common method bias

Regarding statistical control, the study employed Harman’s one-factor test, and exploratory factor analysis was conducted on all items of the research variables. The results revealed that five factors had eigenvalues greater than 1, and the variance explained by the first factor was 29.32%, which is below the critical threshold of 40%. Therefore, it could be concluded that there is no severe common method bias in the study.

### 3.4 Mediation analysis

As shown in Table 3, the mediation analysis revealed that family health had a significant total effect on depressive symptoms. The indirect effect of family health on depressive symptoms via stress was also significant, indicating that stress partially mediated this relationship. Specifically, family health was negatively associated with stress, and stress was positively associated with depressive symptoms. The direct effect of family health on depressive symptoms remained significant, further supporting partial mediation. All effects were supported by bootstrap confidence intervals that did not cross zero.

**Table 3:** Mediation analysis (*n* = 1,193).

Variable	*B*	(Boot)SE	(Boot)LLCI	(Boot)ULCI
Total effect	−0.234	0.032	−0.297	−0.171
Indirect effects:	−0.085	0.014	−0.113	−0.059
Path a: Family—Stress	−0.228	0.032	−0.302	−0.176
Path b: Stress—Depressive symptoms	0.355	0.027	0.302	0.408
Direct effect	−0.149	0.031	−0.210	−0.089

### 3.5 Moderated mediation analyses

**Figure 1:**
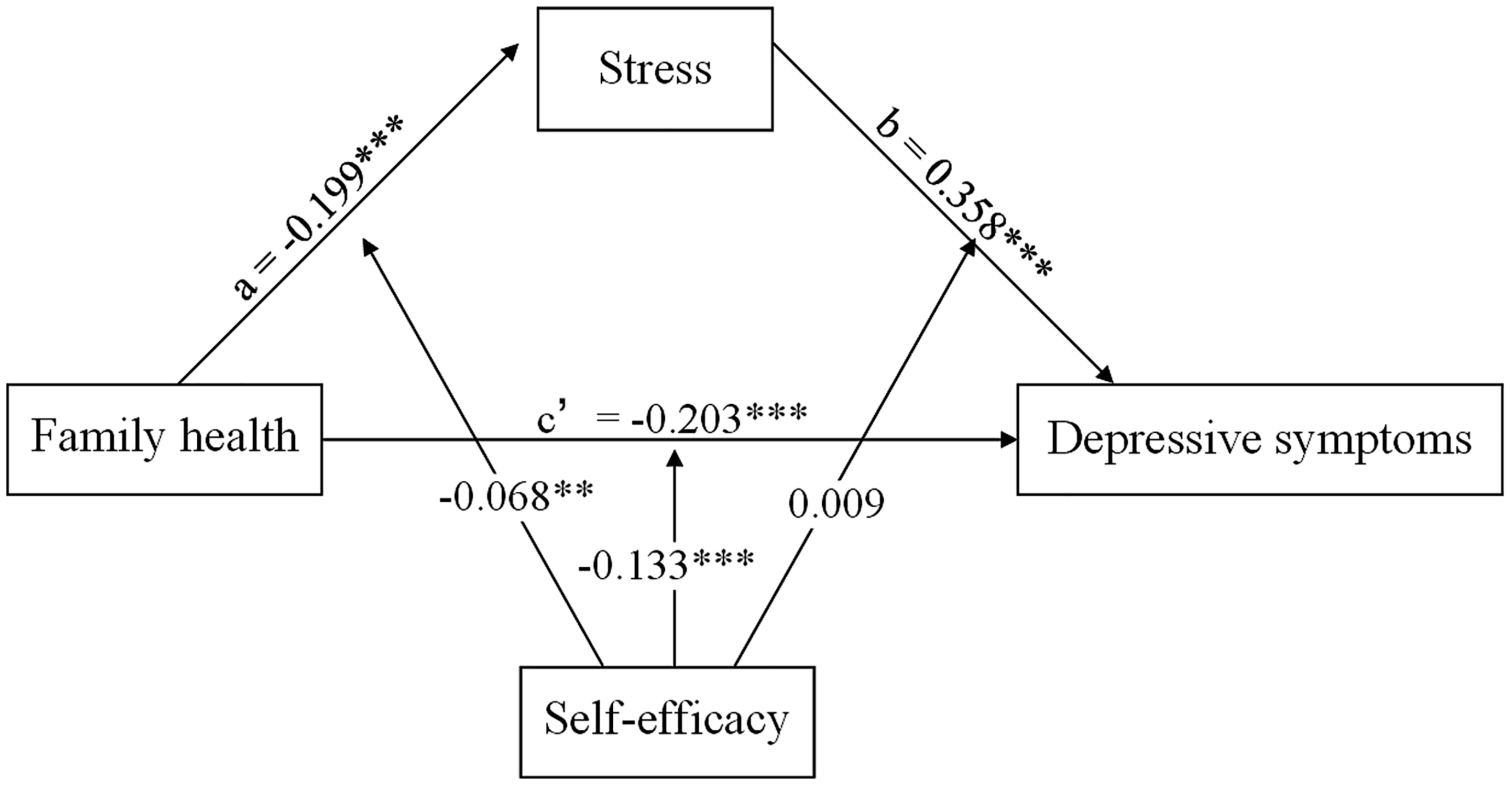
The final moderated mediation model (** p < 0.01, *** p < 0.001).

Table 4 displayed the results of the moderated mediation analysis. Moderated mediation analysis indicates that self-efficacy does not moderate the indirect effect (path b: stress—depressive symptoms) of the mediation model (self-efficacy * stress: *B* = 0.009, 95% CI: −0.04, 0.057). Furthermore, family health played a significant regulatory role in both the direct effect (self-efficacy * family health: *B* = −0.046, 95% CI: −0.083, −0.009) and the indirect effect (path a: family health—stress: self-efficacy * family health: *B* = −0.133, 95% CI: −0.180, −0.086) of the moderated model. Moreover, the final moderated mediation model was depicted in Figure 1.

**Table 4:** Moderated mediation analysis (*n* = 1,193).

Variable	*B*	SE	*t*	LLCI	ULCI
Outcome: stress					
Family health	−0.199	0.035	−5.695***	−0.267	−0.130
Self-efficacy	−0.098	0.033	−2.993**	−0.162	−0.034
Self-efficacy*Family health	−0.068	0.025	−2.736**	−0.116	−0.019
Outcome: depressive symptoms					
Family health	−0.203	0.033	−6.189***	−0.267	−0.139
Stress	0.352	0.027	13.144***	0.299	0.404
Self-efficacy	0.122	0.031	4.009***	0.062	0.182
Self-efficacy*Family health	−0.133	0.024	−5.541***	−0.180	−0.086
Self-efficacy*Stress	0.009	0.025	0.349	−0.040	0.057

** *p* < 0.01, *** *p* < 0.001.

By analyzing the indirect effect of family health on depressive symptoms at different levels of self-efficacy, we further tested the significant moderated mediation model. As shown in Table 5, self-efficacy was categorized into three levels: low (mean minus one standard deviation), medium (mean), and high (mean plus one standard deviation). Specifically, self-efficacy played a significant mediating role in the relationship between family health and depressive symptoms, especially when the self-efficacy level of university students was low, at the medium level, and at the high level.

**Table 5:** Conditional indirect effects of family health on depressive symptoms at values of self-efficacy (*n* = 1,193).

Self-efficacy level	*B*	BootSE	BootLLCI	BootULCI
Low self-efficacy	−0.045	0.016	−0.079	−0.014
Moderate self-efficacy	−0.070	0.015	−0.099	−0.043
High self-efficacy	−0.096	0.022	−0.144	−0.057

**Figure 2:**
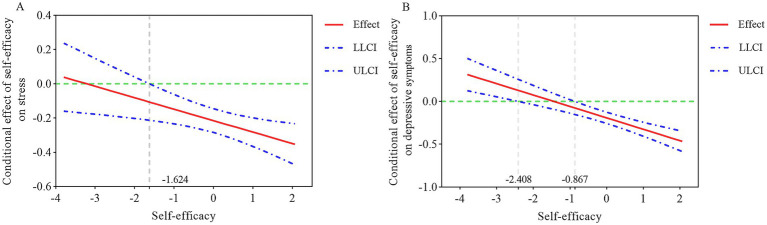
Johnson-Neyman technique for the moderating effect of self-efficacy. **(A)** The conditional effect of family health on stress at the value of self-efficacy. **(B)** The conditional effect of family health on depression at the values of self-efficacy.

Subsequently, the Johnson-Neyman technique indicated that when the standard score of self-efficacy was above −1.624 in Figure 2A, self-efficacy could significantly moderate the indirect effect of family health on depressive symptoms (path a: family health—stress), with the 95% confidence interval not containing zero. Similarly, Figure 2B shows that when the standard score of self-efficacy was below −2.408 or above −0.713, self-efficacy could significantly moderate the direct effect of family health on depressive symptoms, as the 95% confidence interval does not include zero in these regions.

## 4 Discussion

In our study, family health is negatively associated with depressive symptoms among university students, and this association is a complex and multifaceted issue. A supportive family environment can provide emotional support, helping university students better cope with life stressors and academic challenges, thereby reducing the occurrence of depressive symptoms (37). Conversely, if the family is fraught with conflict, violence, or indifference, these negative factors can increase stress levels in university students, leading to feelings of loneliness and helplessness, thus raising the risk of developing depression (37–39). Moreover, economic difficulties, parental divorce, or health issues within the family can also adversely affect the psychological well-being of children (40). Adolescents are in a stage of life where they seek independence and have developed individual thoughts and personalities, resulting in an increased need for autonomy (41). Consequently, conflicts often arise with parents over supervision and control, which will affect the health of family relationships (42). This diminishes their sense of emotional security and hinders the development of their emotional regulation skills, manifesting in more significant emotional and behavioral problems.

Stress partially mediates the relationship between family health and depressive symptoms, revealing the potential mechanisms by which family health indirectly affects depressive symptoms. When family health is favorable, it provides university students with effective support and buffering, helping them better cope with various stressors in life and reducing the occurrence of depressive symptoms. Research indicates that academic stress is one of the primary sources of stress for university students, including exam performance, workload, and future job prospects, which often lead to anxiety and even depression (43). Additionally, high parental expectations regarding academic achievement can further increase students’ stress, making them feel burdened in their pursuit of academic success (44). Negative interactions and conflicts within the family can undermine students’ self-esteem and sense of self-worth, making them more susceptible to stress and increasing the risk of depressive symptoms. In such scenarios, university students tend to withdraw, lacking positive social interactions, which further increases their sense of isolation and psychological stress. This self-imposed isolation and sense of loneliness stem not only from negative interactions within the family but can also extend to broader interpersonal relationships (45). Interpersonal conflicts can activate an individual’s stress response system, including the increased secretion of stress hormones such as cortisol. Studies have shown that prolonged high levels of stress response negatively affect brain structure and function, particularly the hippocampus and prefrontal cortex, which play crucial roles in emotion regulation and cognitive function (46). Reduced hippocampal volume is closely associated with depressive symptoms, while decreased prefrontal cortex function impacts decision-making and self-control, making individuals more prone to negative emotions (47).

Our findings indicated that self-efficacy did not significantly moderate path b, the association between stress and depressive symptoms. We speculate that once individuals experience a high level of stress, its impact on depression may become more direct, limiting the buffering role of self-efficacy. However, self-efficacy significantly moderated both the direct effect of family health on depressive symptoms and path a (family health—stress). This partially supported social cognitive theory, which emphasizes the crucial role of self-efficacy in managing stressors such as poor family functioning. Specifically, individuals with high self-efficacy may be more capable of regulating their stress cognitively and behaviorally when faced with adverse family conditions, thereby reducing the likelihood of developing depressive symptoms. The moderating role of self-efficacy can be explained through its impact on the brain. Research indicates that the prefrontal cortex (PFC) plays a key role in regulating self-efficacy. Higher levels of self-efficacy are associated with increased activity in the PFC, a region responsible for executive functions and decision-making processes, which help individuals plan effectively, inhibit impulses, and solve problems (48). Therefore, when family health is poor, high self-efficacy can enhance the function of the PFC, enabling individuals to better cope with life’s challenges and thus reduce depressive symptoms. Additionally, individuals with high self-efficacy are more likely to experience feelings of accomplishment and pleasure from successfully completing tasks. This positive emotion not only alleviates stress and negative feelings but also, by influencing the brain’s reward system (including the nucleus accumbent and dopaminergic system), further reduces depressive symptoms (49). Regarding the stress response, individuals with high self-efficacy might exhibit different responses in their hypothalamic–pituitary–adrenal (HPA) axis (50), which is the body’s primary stress regulation system (51). High self-efficacy can modulate HPA axis activity, reducing the secretion of stress hormones such as cortisol, thus enhancing emotional regulation and decreasing the incidence of depressive symptoms in the face of stress issues (52). The sense of self-efficacy is closely related to coping strategies and emotional regulation capabilities (53). Individuals with high self-efficacy tend to use positive coping strategies when dealing with stress, such as problem-solving, seeking social support, and positive reappraisal (54). These strategies effectively reduce stress levels and mitigate negative emotions. In contrast, individuals with low self-efficacy are more likely to use negative coping strategies, such as avoidance and denial, leading to stress accumulation and an increased risk of depressive symptoms.

The research findings suggest that when evaluating the mental health status of university students, the interaction of multiple factors must be considered. In addition to family health, stress, and self-efficacy, patterns of neural activity should be regarded as important factors. Future research can further explore the relationship between self-efficacy and neural activity to develop more comprehensive and effective mental health intervention strategies. Evaluating brain activity associated with depression can incorporate various neuroimaging techniques such as near-infrared spectroscopy (NIRS), functional magnetic resonance imaging (fMRI), electroencephalography (EEG), and transcranial Doppler ultrasonography (TCD). These techniques can provide information on neural activity from different dimensions, including oxygen levels, electrical signals, and blood flow velocity, offering a more comprehensive perspective for depression research. By combining multiple neuroimaging techniques, it is possible to investigate the differences in neural activity patterns among different depression patients and the impact of intervention methods on brain structure and function. This can help identify neural targets or intrinsic mechanisms for treating depression in university students, thereby enhancing the specificity and effectiveness of treatment. These studies can not only provide scientific evidence for mental health interventions but also facilitate the development of self-efficacy training methods based on neurofeedback, further improving intervention outcomes.

However, this study has some limitations. Firstly, the study employs a cross-sectional design, which does not account for the influence of temporal factors on the findings. Future research could adopt a longitudinal design to further verify the causal relationships between these variables. Secondly, the ability to implement interventions or control other variables was limited, making it challenging to rule out other confounding factors that might influence the study results. Lastly, the research relies on self-reports or recollections from the respondents, which may lead to memory biases and subjective biases, thereby reducing the accuracy of the data.

## 5 Conclusion

This study provided a new perspective on the relationships between family health, stress, self-efficacy, and depressive symptoms among university students. A key finding is the high prevalence of depressive symptoms during the pandemic. Family health was indirectly linked to depressive symptoms through stress, with self-efficacy partially moderating these pathways. These findings highlight the need for universities and mental health professionals to prioritize student well-being by addressing the interconnected roles of family health, stress, and self-efficacy. Enhancing family involvement and strengthening self-efficacy may help reduce stress and depression, ultimately improving students’ mental health, academic performance, and overall resilience.

## Data Availability

The datasets presented in this study can be found in online repositories. The names of the repository/repositories and accession number(s) can be found at: the data utilized in this study were derived from the Psychology and Behavior Investigation of Chinese Residents (PBICR) survey, https://www.x-mol.com/groups/pbicr.
